# Healthcare professionals' knowledge, attitude and its associated factors toward electronic personal health record system in a resource-limited setting: A cross-sectional study

**DOI:** 10.3389/fpubh.2023.1114456

**Published:** 2023-03-15

**Authors:** Sisay Maru Wubante, Masresha Derese Tegegne, Mequannent Sharew Melaku, Nebyu Demeke Mengiste, Ashenafi Fentahun, Wondosen Zemene, Makida Fikadie, Basazinew Musie, Derso Keleb, Habtemaryam Bewoketu, Seid Adem, Simegne Esubalew, Yohannes Mihretie, Tigist Andargie Ferede, Agmasie Damtew Walle

**Affiliations:** ^1^Department of Health Informatics, Institute of Public Health, College of Medicine and Health Sciences, University of Gondar, Gondar, Ethiopia; ^2^North Shewa Zonal Health Department, Department of Monitoring and Evaluation, Shewa, Ethiopia; ^3^Department of Health Informatics, Bahirdar Health Science College, Bahir Dar, Ethiopia; ^4^South Wollo Zonal Health Department, Akesta Primary Hospital, Akesta, Ethiopia; ^5^South Gondar Zonal Health Department, Nifas Mewocha Primary Hospital, Nefas Mewucha, Ethiopia; ^6^Department of Epidemiology, Institute of Public Health College of Medicine and Health Sciences, University of Gondar, Gondar, Ethiopia; ^7^Department of Health Informatics, College of Health Science, Mettu University, Mettu, Ethiopia

**Keywords:** health professionals, factors, electronics personal health record system, knowledge, attitude, Ethiopia

## Abstract

**Introduction:**

Electronic personal health record (e-PHR) system enables individuals to access their health information and manage it themselves. It helps patient engagement management of health information that is accessed and shared with their healthcare providers using the platform. This improves individual healthcare through the exchange of health information between patients and healthcare providers. However, less is known about e-PHRs among healthcare professionals.

**Objective:**

Therefore, this study aimed to assess Health professionals' Knowledge and attitude and its associated factors toward e-PHR at the teaching hospital in northwest Ethiopia.

**Methods:**

An institution-based cross-sectional study design was used to determine healthcare professionals' knowledge and attitude and their associated factors toward e-PHR systems in teaching hospitals of Amhara regional state, Ethiopia, from 20 July to 20 August 2022. Pretested structured self-administered questionnaires were used to collect the data. Descriptive statistic was computed based on sociodemographic and other variables presented in the form of table graphs and texts. Bivariable and multivariable logistic analyses were performed with an adjusted odds ratio (AOR) and 95% CI to identify predictor variables.

**Result:**

Of the total study participants, 57% were males and nearly half of the respondents had a bachelor's degree. Out of 402 participants, ~65.7% [61–70%] and 55.5% [50–60%] had good knowledge and favorable attitude toward e-PHR systems, respectively. Having a social media account 4.3 [AOR = 4.3, 95% CI (2.3–7.9)], having a smartphone 4.4 [AOR = 4.4, 95% CI (2.2–8.6)], digital literacy 8.8 [(AOR = 8.8, 95% CI (4.6–15.9)], being male 2.7 [AOR = 2.7, 95% CI (1.4–5.0)], and perceived usefulness 4.5 [(AOR = 4.5, 95% CI (2.5–8.5)] were positively associated with knowledge toward e-PHR systems. Similarly, having a personal computer 1.9 [AOR = 1.9, 95% CI (1.1–3.5)], computer training 3.9 [AOR = 3.9, 95% CI (1.8–8.3)], computer skill 19.8 [AOR = 19.8, 95% CI (10.7–36.9)], and Internet access 6.0 [AOR = 6.0, 95% CI (3.0–12.0)] were predictors for attitude toward e-PHR systems.

**Conclusion:**

The findings from the study showed that healthcare professionals have good knowledge and a favorable attitude toward e-PHRs. Providing comprehensive basic computer training to improve healthcare professionals' expectation on the usefulness of e-PHR systems has a paramount contribution to the advancement of their knowledge and attitude toward successfully implementing e-PHRs.

## Introduction

The past two decades have seen much development in information and communication technology applications in healthcare delivery systems (1). Boosting self-care and patient involvement in care management have progressively become key components of efforts to enhance health service delivery and care quality for chronic disease (2). Electronic personal health records (ePHRs) offer a tool to empower patients and self-care advocates (3).

Electronic personal health records can be defined as electronic applications through which individuals can access, manage, and share health information in a private, secure, and confidential environment (4, 5). Existing evidence shows their benefits in improving outcomes, especially for patients with chronic disease (6). Enhanced communication and information exchange among healthcare providers is critical to improve the quality and safety of healthcare and create efficiencies in healthcare systems, facing increasing resource demands as the population ages (7, 8). The most common PHR model is a digital archive of personal health information that patients and others authorized by patients can manage and access (9). The EHR has the potential to sufficiently motivate and engage patients in taking a more active role in managing their health, which has been identified as a critical condition for improving care quality and efficiency (10). As a result, PHRs have been recognized as a tool for patient engagement (11). According to a study done in the USA, 71% of ePHRs open a wide opportunity for healthcare professionals, including retrieving, accessing, and processing patient data, sending automated reminders to avoid medication errors, enhancing health information exchange between healthcare professional and patients, and ensuring complete and legible documentation of the patient's condition (12). Studies in Europe revealed that 80% of ePHRs are used to improve the accessibility and quality of care for patients (13). In Australia, ePHRs are used to boost patient engagement, improve patient–provider information exchange, encourage self-management, and increase empowerment of patients (14). In low-income countries, including Africa, digital personal health information record utilization is low as compared with developed countries (15). Even though digital personal health information record systems have wide applications by encouraging healthcare providers to provide access to their patients, the use of individual e-PHR remains low in low-income countries (16, 17).

Despite the significant opportunities for ePHRs to improve healthcare, there are constraints to the wide utilization of ePHRs (18). According to studies, both patients and providers are interested in ePHRs, especially as a means of increasing patient empowerment. However, there are obstacles to overcome and challenges to face when implementing ePHRs (19). Some of these barriers are related to knowledge, attitude, training in digital literacy, and so on (20, 21).

A study conducted on electronic personal record utilization showed that only 18.9% of individuals use it, which is low (12). According to the results of the survey of nurses' attitudes, 76% indicated that the e-HER system would have a positive effect on improving patient care (22).

A survey study conducted on healthcare professionals' attitudes toward ePHRs showed that the majority of them had a favorable attitude (23). A cross-sectional study conducted on healthcare professionals showed that they had good knowledge of ePHRs (24).

Evidence from Ethiopia suggests that the country's health workforce is under-resourced, and as a result, its population experiences a variety of health issues, such as difficulty accessing specialists and increasing medical costs (25). The e-PHRs can assist in resolving these issues by facilitating prompt access to specialists, lowering associated travel expenses, and decreasing waiting times, such as the financial issues with ICT infrastructure (26).

Even though having an ePHR system was essential for delivering high-quality patient care and enhancing the effectiveness and efficiency of the healthcare system (27), poor implementation can be found in low-income countries (28). The Federal Ministry of Health was currently concentrating on getting the healthcare system computerized to provide timely and evidence-based quality healthcare services (29). This demonstrates the government's strong commitment to a paradigm shift regarding the use of information and communication technology and the use of e-PHRs in healthcare delivery systems (30). The Ethiopian government supports several projects to better meet the population's need for healthcare. One of the four goals of the health sector transformation plan is the information revolution, which is the focus of Ethiopia's Ministry of Health activities. This agenda's success is measured by culture change for decision-making using health data, digitization, and scale-up. Initiatives in health information and communication technology and a national digital health policy were also established for the healthcare system's digitization.

This study sought to evaluate healthcare professionals' knowledge and attitude and their associated factors toward ePHRs. Therefore, the results of the research could serve as a starting point for the development of e-PHR applications and the future success of e-PHR programs in Ethiopia. Policymakers and other stakeholders may use the study results to create a plan based on the findings. The results of this study will also serve as a benchmark for future scholars interested in the subject.

According to our in-depth literature reviews, evidence showed that there is limited research demonstrated in Ethiopia related to knowledge and attitude toward ePHR among healthcare professionals. Therefore, this study determined knowledge and attitude and their associated factors toward ePHR among healthcare professionals. The study results might be used by the Ethiopia's Ministry of Health, teaching hospitals, and different stakeholders to take action before the implementation of the ePHR and to formulate a plan based on the findings. Moreover, the findings of this study were used as a baseline study by upcoming researchers interested in the area.

## Method and materials

### Study design and settings and period

An institution-based cross-sectional study was used to determine healthcare professionals' knowledge and attitude and their associated factors toward ePHRs in the teaching hospital in Amhara Region, Ethiopia, from 20 July to 20 August 2022. Ethical approval was obtained from the institutional review board of the College of Medicine and Health Sciences, University of Gondar with reference number (HI/1161/12/14).

### Study area

The study area was teaching hospitals found in the Amhara region of northwest Ethiopia. The study was conducted at private hospitals in the Amhara region. It has 13 administrative zones and 181 woredas. Amharic is the working language of the state. The capital city of the State of Amhara is Bahir Dar.

### Study participants and sample size determination

The study populations included in this research were all healthcare professionals working at teaching hospitals who have above 6 months of work experience. Those study subjects who have experience in their working environment have information about the research questions. However, healthcare professionals who were severely ill and on annual leave during data collection were excluded.

### Sample size and sampling procedures

The sample size was determined using the single population proportion formula, and the 50% proportion was considered since there had been no previous study conducted on healthcare professionals' knowledge and attitude across the country in similar settings. By considering a 95% confidence interval (CI), 5% marginal error, and 10% non-response rate, the minimum sample size of 423 was obtained. A simple random sampling technique was used to recruit the study participants. The total sample size was proportionally allocated to each profession.

### Study variables and outcome measurements

This study's outcome variables were healthcare professionals' knowledge and attitude toward ePHR systems. Independent variables were age, sex, level of education, profession, monthly income, year of experience, computer training, computer skill, digital literacy, Internet access, smartphone, laptop, and having a social media account.

#### Attitude

It was defined as one's favorable or unfavorable feelings toward the introduction of new types of ePHR technology in any environment. For the attitude questions, study participants who scored above the median value on the 5-point Likert scale were categorized as having a favorable attitude, and those who scored below the median value were categorized as having an unfavorable attitude.

#### Knowledge

It involves knowing the medical applications of ePHR systems, the e-PHRS infrastructure, and the effect of e-PHRS on quality of care, cost, and time. For the knowledge questions, study participants who scored above the median value were categorized as having good knowledge, and those who scored below the median value were categorized as having poor knowledge (12).

#### Digital literacy

Digital literacy was measured by 16-point Likert scale response options ranging from strongly agree to strongly disagree and was defined as the level of technical knowledge on the sharing of personal health information and experiences with electronic resources. Due to the non-normal distribution of the outcome variable, participants who scored above the median values were considered high digital literacy levels and those who scored below the median values were considered low digital literacy levels (31).

#### Computer skill

It is defined as the knowledge that enables you to work with computers and other related technologies, and healthcare professionals have basic computer skill when they scored above the median value and no computer skill when they scored below the median value, which was assessed by using 5-point Likert scale questions (32).

#### Perceived usefulness

It is the degree to which users' perceptions of the health-related services offered by personal health records will be beneficial to them. Study participants who scored higher median value on the 5-point Likert scale were categorized as “thinking personal health records will be useful for their health information management,” and those who scored below the median value were categorized as “thinking mobile personal health record system will not be useful for their health information management” (33).

### Data collection tools and quality control

Pretested, structured, and self-administered questionnaire was used to collect data from study participants. The questionnaires contain sociodemographic, technical, behavioral, organizational, and access-to-basic technology variables. For semantic consistency, the questionnaire was first written in English, then translated into Amharic, and then back into English.

The instrument (the tool) used for the study was obtained from many literary studies, with adjustments made to fit our setting and the goal of the research. It includes yes-or-no questions on a 5-point Likert scale. To control the quality of the data, 2 days of training were given to data collectors and supervisors on the objective of the study, data collection procedures, data collection tools, respondents' approach, and data confidentiality. Before the actual data collection, pretesting of the questionnaire was conducted for about 10% of the study participants outside of the study area. The result of Cronbach's alpha was obtained to show the internal consistency of the questionnaires.

### Data management and analysis

Data entry and cleaning were performed using the Kobo toolbox. Therefore, data were exported to SPSS version 26 for analysis purposes. Descriptive statistics and percentages were computed to describe the sociodemographic characteristics of the study participants and the outcome variable. Bivariable and multivariable logistic regression model analyses were performed to show the association between dependent and independent variables. In bivariable logistic regression analysis, factors with *P* < 0.2 were candidates for multivariable logistic regression analysis. Odds ratios with 95% CI *P*-values were computed to show statistical significance. For statistical test significance, the cutoff value of *P* < 0.05 was considered. The model fitness was checked by the Hosmer–Lemeshow test.

## Result

### Sociodemographic characteristics of the study participant

Of the total 423 recruited study subjects, 402 participants responded to the questionnaires, with a response rate of 95%. More than half of the 229 (57%) participants were males. Of the total respondents, 155 (38.6%) were in the age group of 21–29 years, with a mean age of 32 ± 6.3 SD. In terms of educational status, nearly half of the 197 (49%) had a bachelor's degree. In terms of profession, 37.3% of the respondents were physicians. In terms of work experience, 221 (55%) of study participants had between one and three jobs. More than half (57%) of the study participants have a monthly salary between ETB 6,001 and 8,000. Table 1 provides detailed information about the sociodemographic characteristics of the study participants.

**Table 1:** Sociodemographic characteristics of the study participants working in teaching hospitals in Amhara region, northwest Ethiopia, 2022 (*N* = 402).

**Variables**	**Category**	**Frequency**	**Percentage (%)**
Sex	Male	229	57
	Female	173	43
Age in years	21–29	155	38.6
	30–35	127	31.6
	>35	120	29.9
Educational level	Diploma	74	18.4
	BSC degree	197	49.0
	Masters and above	131	32.6
Types of profession	Physician	150	37.3
	Health officer	27	6.7
	Nurses	107	26.6
	Pharmacy	14	3.5
	Midwives	79	19.7
	Radiology	6	1.5
	Anesthesia	12	3.0
	Psychiatry	7	1.7
Working experience	1–3years	221	55.0
	4–7 years	70	17.4
	8–10 years	23	5.7
	>10 years	88	21.9
Monthly income	3,333–6,000	130	32.3
	6,001–8,000	229	57.0
	>8,001	43	10.7

### Access to basic technologies

Of the total 402 study participants, 376 (93.5%) had never used ePHRs previously. More than 217 (54%) of respondents had a private computer. Of the total study subjects, 283 (70.4%) had a smartphone, and more than two-thirds, i.e., 269 (69.9%) had social media accounts (Table 2).

**Table 2:** Access to basic technologies of the study participants working in teaching hospitals in the Amhara region, northwest Ethiopia, 2022 (*N* = 402).

**Variables**	**Category**	**Frequency**	**Percentage (%)**
Do you have used e-PHRS previously	No	376	93.5
	Yes	26	6.5
Do you have a personal computer	No	185	46.0
	Yes	217	54.0
Do you have a smartphone	No	119	29.6
	Yes	283	70.4
Do you have social media account	No	133	33.1
	Yes	269	69.9

### Technical factors of study participants

Of the total of 402 study subjects, 259 (64.4%) perceived the usefulness of ePHRs for managing their health. Nearly two-thirds of participants had computer skill, and about 60.4% of study participants were digitally literate (Table 3).

**Table 3:** Technical factors of the study participants working in teaching hospitals in the Amhara region, northwest Ethiopia, 2022 (*N* = 402).

**Variables**	**Category**	**Frequency**	**Percentage (%)**
Perceived usefulness	Not useful	143	35.6
	Useful	259	64.4
Computer skill	No	151	37.6
	Yes	251	62.4
Computer training	No	97	24.1
	Yes	305	75.9
Digital literacy	No	159	39.6
	Yes	243	60.4

### Organizational factors

Of the total respondents, about 194 (483%) had a computer available in their office. More than two-thirds (79.1%) of study participants had Internet access at their offices. Regarding IT support staff, 255 (68.4%) of participants indicated that support personnel is available in their hospital (Table 4).

**Table 4:** Organizational factors of the study participants working in teaching hospitals in the Amhara region, northwest Ethiopia, 2022 (*N* = 402).

**Variables**	**Category**	**Frequency**	**Percentage (%)**
Computer available in your office	No	208	51.7
	Yes	194	48.3
Internet access in your office	No	84	20.9
	Yes	318	79.1
The organization has IT support staff	No	127	31.6
	Yes	255	68.4

### Study participants' knowledge of e-PHRS

**Figure 1:**
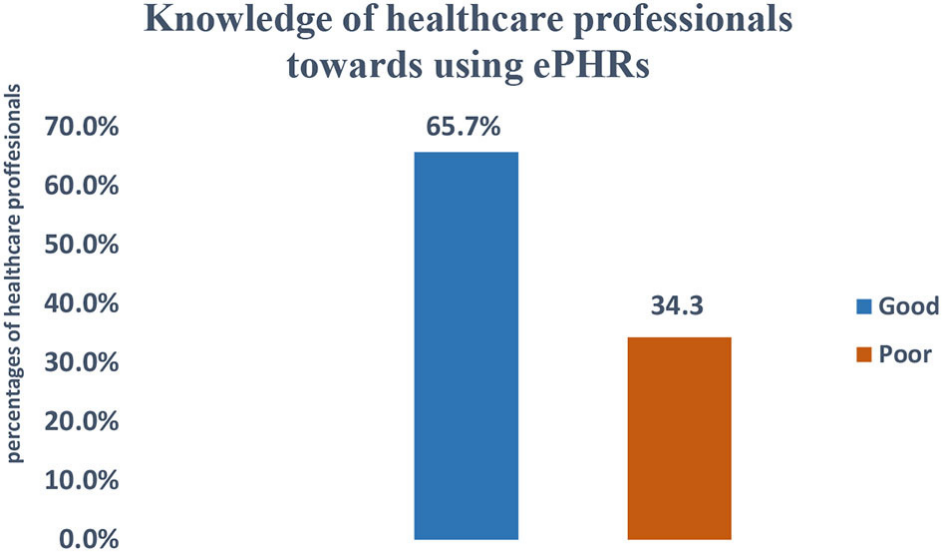
Knowledge of e-PHRS study participants working at teaching hospitals in Amhara region, northwest Ethiopia, 2022 (*N* = 402).

Of the total study participants, 65.7% [61–70%] had good knowledge of electronic personal health records (Figure 1).

### Study participants' attitudes toward e-PHRS

**Figure 2:**
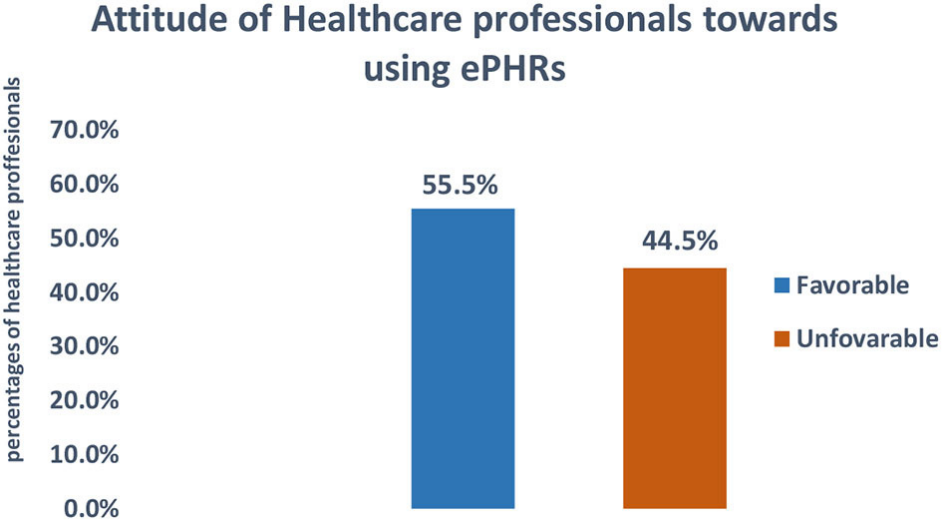
Attitude toward e-PHRS study participants working in teaching hospitals in Amhara region, northwest Ethiopia, 2022 (*N* = 402).

Of the total respondents, 55.5% [50–60%] of respondents had a favorable attitude toward the ePHR system (Figure 2).

### Factors associated with study participants' knowledge of e-PHRS

As shown in Table 5, from the total variables entered into the bivariable logistic regression analysis, social media account, smartphones, digital literacy, perceived usefulness, sex, and Internet access were factors associated with knowledge of e-PHRS in bivariable analysis at a *p*-value < 0.02. Those variables were drawn into a multivariable logistic regression model to control confounders.

**Table 5:** Multivariable logistic regression analysis on factors associated with knowledge toward e-PHRS among healthcare professionals in the Amhara region, northwest Ethiopia 2022 (*N* = 402).

**Variables**	**Knowledge**		**Crude odds ratio (95% CI)**	**Adjusted odds ratio (95% CI)**
	**Good**	**Poor**		
**Social-media account**				
Yes	219 (81.4%)	50 (18.6%)	8.6 (5.4–13.7)	4.3 (2.3–7.9)
No	45 (33.8%)	88 (66.2%)	1	
**Have smartphone**				
Yes	221 (78.1%)	62 (21.9%)	6.3 (3.9–10.0)	4.4 (2.2–8.6)
No	43 (36.1%)	76 (63.9%)	1	
**Digital literacy**				
Yes	213 (87.7%)	30 (12.3%)	15 (9.5–24.9)	8.8 (4.6–15.9)
No	51 (32.1%)	108 (67.9%)	1	
**Perceived usefulness**				
Yes	207 (79.9%)	52 (20.1%)	6.0 (3.8–9.4)	4.5 (2.5–8.5)
No	57 (39.9%)	86 (60.1%)	1	
**Sex**				
Male	164 (71.6%)	65 (28.4%)	1.8 (1.2–2.8)	2.7 (1.4–5.0)
Female	100 (57.8%)	73 (42.2%)	1	
**Internet access**				
Yes	227 (71.4%)	91 (28.6%)	3.2 (1.9–5.2)	4.8 (2.3–9.9)
No	37 (44.%)	47 (56%)	1	

Having a social media account was found to be positively associated with the study participants' knowledge of e-PHRS. Those study participants who had social media accounts were 4.3 [AOR = 4.3, 95% CI (2.3–7.9)] times more likely to know e-PHRS as compared with their counterparts.

Having a smartphone was significantly associated with knowledge of e-PHRS. Smartphone owners were 4.4 [AOR = 4.4, 95% CI (2.2–8.6)] times more likely to know those who do not have a smartphone.

Digital literacy was positively associated with knowledge of e-PHRS. Those study subjects who had digital literacy were 8.8 [(AOR = 8.8, 95% CI (4.6–15.9)] times more likely to have knowledge of e-PHRS than their equivalents.

Sex was found to be statistically significant with knowledge of e-PHRS. Male study participants were 2.7 [AOR = 2.7, 95% CI (1.4–5.0)] times more likely to have knowledge of e-PHRS than females.

Perceived usefulness was significantly associated with knowledge of e-PHRS. Those study participants' beliefs on the usefulness of the e-PHRS were 4.5 [(AOR = 4.5, 95% CI (2.5–8.5)] times more likely to have knowledge of e-PHRS than their counterparts.

### Factors associated with study participants' attitudes toward e-PHRS

As shown in Table 6, from the total variables entered into bivariable logistic regression analysis, having a social media account, having a computer, computer training, computer skill, and Internet access were factors associated with attitude on e-PHRS in bivariable analysis at a *p*-value < 0.02. Those variables were drawn into a multivariable logistic regression model to control confounders.

**Table 6:** Multivariable logistic regression analysis on factors associated with attitude toward e-PHRS among healthcare professionals in the Amhara region, northwest Ethiopia 2022 (*N* = 402).

**Variables**	**Attitude**		**Crude odds ratio (95% CI)**	**Adjusted odds ratio (95% CI)**
	**Favorable**	**Unfavorable**		
**Have computer**				
Yes	149 (68.7%)	68 (31.3%)	3.4 (2.3–5.0)	1.9 (1.1–3.5)
No	73 (39.5%)	112 (60.5%)	1	
**Computer training**				
Yes	205 (67.2%)	100 (32.8%)	9.6 (5.4–17.2)	3.9 (1.8–8.3)
No	17 (17.5%)	80 (82.5%)	1	
**Computer skill**				
Yes	201 (80.1%)	50 (19.9%)	24.8 (14.3–44.4)	19.8 (10.7–36.9)
No	21 (13.9)	130 (86.1%)	1	
**Internet access**				
Yes	199 (62.6%)		4.4 (2.6–7.5)	6.0 (3.0–12.0)
No	23 (27.4%)		1	
**Social media account**				
Yes	160 (59.5%)	109 (40.5%)	1.68 (1.1–2.5)	0.92 (0.5–1.7)
No	62 (46.6%)	71 (53.4%)	1	

Having a personal computer was found to be positively associated with study participants' attitudes toward e-PHRS. Those study participants who had personal computers were 1.9 [AOR = 1.9, 95% CI (1.1–3.5)] times more likely to have a favorable attitude toward e-PHRS as compared with their counterparts.

Computer training was significantly associated with attitude toward e-PHRS. Study subjects who had computer training were 3.9 [AOR = 3.9, 95% CI (1.8–8.3)] times more likely to have favorable attitudes than their counterparts.

Computer skill was found to be significantly associated with attitude on e-PHRS. Those study subjects who had computer skill were 19.8 [AOR = 19.8, 95% CI (10.7–36.9)] times more likely to have a favorable attitude than those study participants with inadequate computer skill.

Internet access was found to be statistically significant with attitude toward e-PHRS. Study subjects who had Internet access were 6.0 [AOR = 6.0, 95%CI (3.0–12.0)] times more likely to have a favorable attitude toward e-PHRS than their counterparts.

## Discussion

### Principal findings

A cross-sectional study aimed to assess healthcare professionals' knowledge and attitude and their associated factors toward ePHRs in resource-limited settings. As far as we know, this is the first study in Ethiopia to look into healthcare professionals' knowledge and attitude toward ePHR systems.

According to this study, two-thirds (65.7%) of healthcare professionals were knowledgeable about using an ePHR system. The possible explanation for the high level of knowledge in this study could be the global expansion of digital technologies, which simplify human workloads and have a positive impact on our day-to-day activities, making it important for healthcare professionals to have a good understanding of technology (34). Another reason could be that healthcare professionals are exposed to smart technologies such as smartphones, laptops, and connective technologies, which allows them to have more information about each individual who can manage their health information through digital platforms (35).

The study showed that more than half (55.2%) of healthcare professionals had a favorable attitude toward ePHR systems. The possible explanation for a favorable attitude toward ePHR systems could be that the benefits of digital technologies outweigh the drawbacks, which can influence healthcare professionals to integrate the ePHR system into their routine activities (36). Another reason is that the expansion of the digital ecosystem may change people's perceptions of digital technologies' applications in healthcare systems that improve the quality of care and the exchange of health information at any time and place for evidence-based decision-making (37).

According to the multivariable logistic regression model, respondents' sex, social media account, digital literacy, perceived usefulness, and ownership of a smartphone were all positively associated with knowledge. Similarly, computer skill, having a personal computer, Internet access, and computer training were significantly associated with an attitude toward e-PHRS.

The study found that healthcare professionals who had social media accounts were more likely to know about ePHRs compared to those who did not. The possible reason that social media have a great potential is to obtain updated information regarding innovative digital health technologies all over the world. This creates awareness about the use and benefit that we gain from using digital health technologies in our healthcare delivery systems and improves healthcare services of individuals and organizations.

The present findings also revealed an association between digital literacy and healthcare professionals' knowledge of ePHR systems.

The possible reason might be that people who are digitally health literate (those who can obtain and apply knowledge from electronic sources to solve a health problem) are better able to manage their health and care issues. Better prevention models can be created, and healthy behaviors can be encouraged.

This study claimed that perceived usefulness was significantly associated with healthcare professionals' knowledge of e-PHRS.

This study claimed that having a smartphone was positively associated with knowledge of e-PHRS. This is due to having smartphones that may be exposed to information about digital technologies. They can search for and understand new emerging technologies. Since the system needs smartphone availability, they are not challenged.

The current study suggested that sex was positively associated with healthcare professionals' knowledge of ePHR systems.

This study revealed that a personal computer was significantly associated with knowledge of ePHR systems. Those study participants who had personal computers were more likely to have a favorable attitude toward e-PHRS as compared with study participants who did not. The possible reason might be that owning a personal computer helps understand how technology simplifies our day-to-day operations. Similarly, the health sector might simplify the workload. Having technology in people's hands might provide access to know other technologies.

The present study showed that computer skill was statistically significant with attitude toward e-PHRS. Computer-skilled study participants were more likely to have a favorable attitude toward the system. Reports supported that computer skill enhances respondents to enjoy the system without any difficulty.

Another determinant of healthcare professionals' attitudes toward ePHRs was found to be Internet access. Those respondents who had Internet access were more likely to have favorable attitude. This might be because the Internet influences access to new advanced technologies and applications in the healthcare system. Internet exposure can impact daily life of humans. In contrast, poor Internet access could deny learning new technology from a far distance. In addition, justification may be that access to Internet technology has offered a medium for technological engagement and enhanced digital communication among healthcare providers all over the world.

This study also indicated that taking basic computer training was found to have a significant association with the attitude of healthcare professionals toward the ePHR system. This could be attributed to the fact that computer-related training is much more likely to improve healthcare professionals' understanding of digital platforms. We speculate that a possible reason for this could be computer training is more likely to increase participant familiarity using technologies. In addition, the explanation might be that training and education usually change people's views and upgrade their knowledge levels and perceptions. Knowing the updated technology have passionate health professionals to engage in newly adopted technologies. Another possibility is that well-organized training would have the best way to teach providers how to approach the challenges and opportunities inherent in the personal health record system. Finally, formal training might enhance significant contribution to healthcare.

### Related works

The number of studies focusing on ePHR systems has increased globally, and although a substantial amount of research on this topic has recently been published, we are aware of no studies that report healthcare professionals' knowledge and attitude and their associated factors toward ePHR systems in resource-limited settings such as Ethiopia.

The World Health Organization has already noted that investment in resources, effective approaches, appropriate assessment measures, and digital healthcare systems is essential for the success of healthcare system transformation to achieve universal health coverage in all countries around the world (38). Surprisingly, we noticed that knowledge and attitude were the most common shortcomings of digital healthcare system implementations and sustainability (39). Previous research on the determinants of digital health implementation in integrated care in low-income countries found that, while the importance of digital health is well-understood, the maturity of its implementation remains low at the moment, due to low knowledge and attitude which is consistent with our findings (40).

Studies on healthcare professionals' knowledge of ePHR systems conducted in Uganda (68%) and Saudi Arabia (64.5%) concluded that they had good knowledge of these systems and could improve patient safety and care quality (41, 42). These findings are congruent with ours since knowledge and the best determination of what is necessary to enable positive changes in the digital healthcare systems to be implemented and sustainable in the future are highlighted as the most determinative of digital health adoption shortcomings.

Nevertheless, the current study finding is lower than that of study on healthcare professionals' knowledge of ePHR systems conducted in Nigeria (79.2%) (42). The possible variations between the current study and Nigeria might be due to the information communication utilization level in Nigeria being high, while Ethiopia is at the infancy stage (43). Other reasons could be low Internet penetration in Ethiopia and low infrastructure fulfillment.

Studies conducted on healthcare professionals' attitudes toward ePHR systems in Turkey (56.4%) and Canada (59.2%) showed that they had a favorable attitude toward the system (44, 45). Those findings were concurrent with ours and attitude is a key influence on the implementation of ePHR systems.

Previous studies on the determinants of healthcare professionals' knowledge toward ePHR systems found that having a social media account had a positive influence on increasing understanding (24, 46). This finding is concurrent with the current study conducted.

The previous studies suggested that healthcare professionals who had digital literacy were more likely to know about ePHR systems (47–49). In the present study, being digitally literate was positively associated with knowledge of ePHR systems.

Studies reported that healthcare professionals who had well-perceived usefulness of the system were more likely to have good knowledge of e-PHRS as compared with their counterparts (8, 50–52). The present finding is under the above findings. This might be due to the fact that individual's awareness of the usefulness of new innovative digital technologies will increase the desire to use personal health record technology.

Studies conducted on healthcare professionals' knowledge of ePHR systems showed that smartphone-owner study participants had good knowledge (53, 54). Correspondingly, our study suggested that having a smartphone was positively associated with knowledge.

A previously conducted study on healthcare professionals' knowledge toward ePHR systems reported that males were more likely to have a good knowledge level (55). Our recent study also reported that males were more likely to have a good level of knowledge of ePHR systems.

A study was conducted on healthcare professionals' attitudes toward ePHR systems before concluding that those respondents who had good personal computers were more likely to develop favorable attitudes (48). Our current finding is concurrent with the above report that personal computer owner respondents were more likely to have a favorable attitude toward the implementation of ePHR systems in the healthcare system.

Studies conducted on healthcare professionals' attitudes toward ePHR systems reported that computer skill was none of the determinant factors of favorable attitudes. Computer skill personnel would have a more likely favorable attitude toward ePHRs (49, 56). The present study consistently supports this result.

Scientific evidence showed that healthcare professionals who had access to the Internet had a favorable attitude toward ePHR systems. Respondents with the ability to access the Internet had a positive or favorable attitude compared with their counterparts (57). Our current finding stated that access to the Internet increases favorable attitudes toward a system.

Furthermore, studies conducted on healthcare professionals' attitude toward the implementation of digital personal health record systems showed that computer training had a positive influence to have a favorable attitude (58, 59). The current finding is concurrent with the previous studies reported.

### Strength and limitation

This study has several strengths. To the best of our knowledge, this is the first study focusing on knowledge and attitude and their determinants among healthcare professionals in resource-limited settings. The finding from this study provides valuable information about the knowledge and attitude of healthcare professionals toward ePHR system in resource-limited countries. This is an institution-based cross-sectional study, and only healthcare professionals who came during data collection were included.

## Conclusion

In general, healthcare professionals had good knowledge and favorable attitude toward ePHR systems. Being male, having a social media account, perceived usefulness, digital literacy, and having a smartphone were positively associated with knowledge of ePHR systems; likewise, Internet access, computer skill, computer training, and having a personal computer were significantly associated with a positive attitude toward ePHR systems. Capacity building of healthcare professionals through providing comprehensive basic computer training and improving healthcare professionals' expectations on the usefulness of ePHR systems have a paramount contribution to the advancement of their knowledge and attitude to implement ePHRs successfully. Furthermore, the present study finding will use baseline data for policymakers' program implementers which are used to draft the design and implement the system as well as baseline evidence for interventional studies.

### The implication of the research

Our study provides a ground overview proportion of knowledge and attitude and their determinants in ePHR systems from healthcare professionals' perspective and that highlights the level of digital maturity discrepancies. Future studies should focus on the impact of other specific dimensions on the implementation of the ePHR systems.

## Data availability statement

The original contributions presented in the study are included in the article/Supplementary material, further inquiries can be directed to the corresponding author.

## Ethics statement

Ethical clearance was obtained from the Institutional Review Board (IRB) of the University of Gondar College of Medicine and Health Sciences, Institute of Public Health with reference number (HI/1161/12/14). Moreover, informed written consent was obtained from the study participants.

## Author contributions

SW: conceptualization, data curation, funding acquisition, investigation, and supervision. SW and MT: formal analysis. SW and AW: methodology. SW and MM: validation. SW and NM: visualization. SW, MF, and BM: writing—original draft. SW, DK, HB, SA, SE, YM, and AF: writing—review and editing. All authors contributed to the article and approved the submitted version.
