# Supplementary material for: Healthcare professionals' knowledge, attitude and its associated factors toward electronic personal health record system in a resource-limited setting: A cross-sectional study
Source: Front Public Health. 2023 Mar 15;11:1114456. doi: 10.3389/fpubh.2023.1114456 (PMC10050470; doi:10.3389/fpubh.2023.1114456)
Supplement: Supplementary file 1 [file Table_1.pdf]

Questionnaires

Part1 socio demographic

No.	Question	Response Option	Code	Skip
1	Gender	1. Male<br>2. Female		
2	Age	______ In a year		
3	Professions	1. Medical Doctor<br>2. Health officer<br>3. Nurse<br>4. Midwifery<br>5. Pharmacy<br>6. Medical Laboratory<br>7. Radiology<br>8. Anesthesia<br>9. Optometry<br>10. Psychiatry<br>Other __……(Specify)_____		
4	Educational Status	1. Diploma<br>2. Bachelor Degree<br>3. General Practitioners<br>4. Specialty (MD+)<br>5. Master's Degree<br>6. Others ……(Specify)		
5	Monthly salary	________in birr		
6	What is your work experience (years)?	_______In Years		

7	Do you have your own Computer/laptop	1. Yes<br>2. No		
8	Do you have your Smartphone	1. Yes<br>2. No		
9	Have you took basic computer trainings	1. Yes<br>2. No		
10	Do you have use Electronic Personal Health Record experience before know?	1. Yes<br>2. No		
11	Do you have to use social media?	1. Yes<br>2. No		

**Instruction:** ***Please try to answer questions by encircling the number*** **that best reflects your computer skill 1= Strongly Disagree 2=Disagree 3=Neutral 4=Agree 5=Strongly Agree**						
201	Can you properly turn on and shut down a computer by yourself?	1	2	3	4	5
202	Are you able to manipulate basic Microsoft Office, Microsoft Office Excel, and Microsoft Office PowerPoint by yourself?	1	2	3	4	5
203	Are you able to search, download information from The internet by yourself?	1	2	3	4	5
204	Can you manipulate Cut, Copy, Past digital data Like Text, Images, Audio, and Video by yourself?	1	2	3	4	5
205	Have you able to send digital data like text images, video audio?	1	2	3	4	5

Part 2: Digital literacy assessing questioner

S.no	Question	Strongly agree	Agree	Neutral	Disagree	Strongly disagree
201	I can search online information using search Engines.					
203	I know that not all online information is reliable					
204	I can communicate with other using digital devices (e.g. Send and receive e-mails)					
205	I am aware that when using digital tools, certain communication rules apply (e.g. sharing personal information).					
206	I can share files with others through simple technological means.					
207	I know that technology can be used to interact with services and I passively use some(e.g. mHealth,e-banking)					
208	I can collaborate with others using traditional technologies.					
209	I can create or delete text, tables, and images by using application software's.					
2010	I have abilities to edit files that are created by other person.					
2011	I can apply and modify simple functions and settings of software and applications that I use					

2012	I can take basic steps to protect my computer (e.g. using anti-viruses and passwords) to protect patient data from unauthorized user.					
2014	I know I should not post private information online					
2015	I am aware that my credentials (username and password) can be stolen.					
2016	I know that using digital technology too extensively can affect my health.					
2017	I can find support and assistance when a technical problem occurs or when using a new device, program or application.					
2018	I have ability to solving routine hardware and software problems encountered while using digital devices.					
2019	I know that digital tools can help me in solving problems. I am also aware that they have their limitations.					
2020	I can install application software's to computers and smartphone.					
2021	I am aware that I need to update my digital skills regularly					

Part3:- Organizational Factors Related Question		
301	Is their computer available at your office	1. Yes<br>2. No
303	Do you think you have internet access in your office?	1. Yes<br>2. No

304	Do you think that your organization has IT support staff?	1. Yes<br>2. No

**Instruction:** ***Please try to answer questions by encircling the number*** **that best reflects your perceived usfulness 1= Strongly Disagree 2=Disagree 3=Neutral 4=Agree 5=Strongly Agree**						
201	Electronic personal health record Would be useful for good health care practice?	1	2	3	4	5
202	Electronic personal health record improved enable to obtain information about the disease	1	2	3	4	5
203	Electronic personal health record helped me manage my health condition effectively	1	2	3	4	5
204	Using Electronic personal health record improves my clinical performance	1	2	3	4	5

PART II:-Knowledge towards electronic personal health record

**Instruction:** ***Please try to answer questions by encircling the number that reflects your level of agreement 1. Yes, 2. No***				
No.	Question	Response question	Code	Skip
1	Do you heard about electronic personal health record system?	1. Yes<br>2. No		
2	Communication between patients and doctors is possible through an electronic personal health record system?	1. Yes<br>2. No		
3	Patients' examination and investigation and other can be recorded through an electronic personal health record system?	1. Yes<br>2. No		

4	Electronic personal records of patients' registration can be maintained through electronic personal health record?	1. Yes<br>2. No		
5	Electronic Personal Health Record can be used record vita signs of patient at any time	1. Yes<br>2. No		
6	Electronic Personal Health Record service can reduce unnecessary referrals and transportation costs?	1. Yes<br>2. No		
7	Electronic Personal Health Record system is important on public health issues like chronic disease management and communicable and non-communicable?	1. Yes<br>2. No		
8	Computer technology, Telecommunication technology & Health care technology can be used in electronic personal health record practice?	1. Yes<br>2. No		
9	Store & Forward method is an approach to delivering telemedicine services through email?	1. Yes<br>2. No		
10	The real-time/live conference method is an online approach to deliver Electronic Personal Health Record services.	1. Yes<br>2. No		
11	Electronic Personal Health Record application is important for remote patient care and management especially elders and disabled?	1. Yes<br>2. No		
12	Electronic Personal Health Record is relevant to educate patients and health professionals on managing health problems?	1. Yes<br>2. No		
13	Electronic Personal Health Record can improve the quality and accessibility of health care services?	1. Yes<br>2. No		

14	We can consulates with a senior physician through electronic personal health record system?	1. Yes<br>2. No		
15	The remote diagnosis and treatment of a patient through personal health record technology?	1.Yes<br>2. No		
16	Electronic personal health record is handled by the patient its self			

Part III:-Attitude related questions towards electronic personal health record

**Instruction:** *Please try to answer questions by encircling the number* that best reflects your attitude. 1= Strongly Disagree 2=Disagree 3=Neutral 4=Agree 5=Strongly Agree						
1	I believe electronic personal health record Reduces medical errors?	1	2	3	4	5
2	I believe electronic personal health record Facilitates the diagnosis and treatment of patients?	1	2	3	4	5
3	I believe electronic personal health record Increase communication between patient and health professionals	1	2	3	4	5
4	I believe electronic personal health record can reduce the number of visits to health care centers?	1	2	3	4	5

5	I believe electronic personal health record enables me to accomplish my task more quickly?	1	2	3	4	5
6	I believe electronic personal health record Provides more comprehensive health care services?	1	2	3	4	5
7	I believe electronic personal health record Improves clinical decisions?	1	2	3	4	5
8	Electronic Personal Health Record is completely compatible with my current situation?	1	2	3	4	5
9	In my opinion, Electronic Personal Health Record secure information confidentiality and patient privacy?	1	2	3	4	5
10	I believe trying electronic personal health record applications is a great opportunity?	1	2	3	4	5
